# A Study of Correlates of Age-Related Macular Degeneration in Patients Attending a Tertiary Hospital

**DOI:** 10.7759/cureus.27443

**Published:** 2022-07-29

**Authors:** Kervi Mehta, Sachin Daigavane

**Affiliations:** 1 Department of Ophthalmology, Jawaharlal Nehru Medical College, Datta Meghe Institute of Medical Sciences University, Wardha, IND

**Keywords:** age-related macular degeneration, diastolic blood pressure, cigarette smoking, risk factors, drusen

## Abstract

Background

Age-related macular degeneration (ARMD) is a dreadful vision disease mainly affecting older people and causing permanent blindness if it remains undiagnosed and untreated. This study is particularly carried out to fill the gaps in the literature regarding the association of various systemic and environmental factors with ARMD.

Objective

We aim to study the correlates of age-related macular degeneration in patients attending a tertiary hospital.

Methods

This study is a hospital-based observational case-control study by nature with 260 participants included of ages more than 45 years. The participants were evaluated for risk factors after proper consenting.

Result

Age (56 years), diastolic blood pressure (DBP) of 80 mmHg, smoking cigarette for more than 10 years, sedentary lifestyle, body mass index (BMI) > 29.9 kg/m^2^, sunlight exposure of more than eight hours, and higher lipid levels are determinants of ARMD.

Conclusion

Smoking is the strongest risk factor associated with ARMD, followed by diastolic blood pressure and body mass index. Lipid levels and a sedentary lifestyle have a weaker correlation with ARMD. However, age and gender are the most important determinants among non-modifiable factors.

## Introduction

Age-related macular degeneration (ARMD) is a long-standing disorder of the fovea and is the most common cause of blindness in the old age population. It is estimated that a total of 30-50 million population are suffering from ARMD in the whole world, and this number is rising dramatically as age advances [1]. It is divided into two types: dry and wet ARMD. Dry or early ARMD is characterized by the presence of drusen, which are lipid and protein depositions between the retinal pigment epithelium (RPE) and the Bruch membrane and may be associated with retinal pigmentary changes. Early ARMD is asymptomatic, and late ARMD or wet ARMD is a vision-threatening disorder that leads to choroidal neovascularization into the retina. Dry ARMD is more prevalent than wet ARMD in India [2]. As claimed by the World Health Organization, 196 million people will have ARMD in 2020, and 288 million people will suffer from ARMD by 2040 from the current 196 million people if it remains uncontrolled and untreated [3]. India has 23.5% of global blindness [4], which suggests that the surveillance of various risk factors and controlling them is the need of the hour so that we can prevent this disease at an early stage before causing significant visual impairment.

ARMD is a multifactorial disease, and various systemic, genetic, and ocular risk factors are associated with ARMD. Among systemic risk factors, smoking, alcohol drinking, body mass index (BMI), lipid levels, lifestyle, and sunlight exposure are the causative factors. ARMD causes central irreversible vision loss in the older population, which causes disability and significantly affects the quality of life [5]. Very less studies have been conducted in this region on the correlates of ARMD; therefore, we intend to study the correlates of ARMD in patients attending the eye outpatient department with the hypothesis that there is a correlation between age, gender, hypertension, smoking habits, alcohol drinking, dietary factors, lifestyle, lipid profile, and sunlight light exposure, and ARMD. It is important to understand this visually dreadful disease and its correlation with various risk factors so that we can suggest preventive methods to control disease progression.

Early ARMD is characterized by drusen size > 63 microns but less than 125 microns with no pigmentary abnormalities present. Intermediate ARMD is characterized by a drusen size of more than 125 microns with pigmentary abnormalities. Late ARMD is characterized by neovascular ARMD and/or geographic atrophy; the presence of these changes within two disc diameters of the fovea in either eye [6]. The presence of intermediate drusen with pigmentary abnormalities has very little risk of developing late ARMD, and its progression increases within five years. The presence of large drusen is associated with a 13% risk of progression to late ARMD [7].

The pathogenesis of ARMD is still unknown, and various studies conducted show that it is because of a complex interaction between environmental, genetic, and personal factors [8]. As age increases, there occurs an accumulation of waste products containing lipofuscin in retinal pigment epithelium cells, superadded by the dysfunction of the Bruch membrane, which causes increased permeability of this membrane, thereby causing the deposition of lipid and proteins between the retinal pigment layer and the Bruch membrane. The death of RPE cells, which eventually occurs, leads to the death of photoreceptor cells, which is the reason behind visual loss, which occurs as time passes by. Some studies suggest molecular factors as a cause of ARMD, demonstrating C-reactive protein (CRP) deposition in complement factor H individuals [9].

## Materials and methods

In this hospital-based observational case-control study, we recruited 260 subjects, of which 130 are ARMD cases and 130 are controls. This two-year study was conducted where patients of age more than 45 years and presence of drusen measuring more than or equal to 63 microns on fundoscopy were taken into inclusion after obtaining approval from the ethical committee of the hospital. The size of drusen was calculated by comparing half of the vein diameter at the optic disc margin by a single vitreoretinal surgeon. Patients with glaucoma, central serous retinopathy, diabetic retinopathy, hypertensive retinopathy, uveitis, and anterior segment pathology impairing fundus examination and those not giving valid consent were excluded from this study. The study adhered to the tenets of the Declaration of Helsinki and was approved by the Institutional Ethical Committee (IEC). The ocular examination included best-corrected visual acuity, slit-lamp examination, intraocular pressure (IOP) measurements, fundoscopy, and slit-lamp biomicroscopy with a 90D lens. General examination was done for all patients documenting body mass index (BMI) and hypertension, and blood lipid levels were assessed. A thorough history-taking was conducted on all participants regarding smoking, alcohol drinking, sunlight exposure, and physical activity using a standard protocol provided in the study. Retinal photographs were taken for documentation. The sample size was calculated based on the Krejcie and Morgan formula [10]. Statistical analysis was conducted using a chi-squared test, z-test for the difference between two means, odds ratio (OR), and multivariate regression analysis, and the software used in the analysis was SPSS version 27.0 (IBM Corp., Armonk, NY, USA).

## Results

The prevalence of ARMD in the subjects ranging in age from 45 to 55 years was observed to be 7.69%, and its prevalence increased from this to 37.69% in ages ranging from 66 to 75 years. The number of subjects increased from 45 years, and the majority fall in the age group of 66-75 years. The mean ages of the subjects with and without ARMD were 65.80±6.72 and 65.17±7.42 years, respectively. The prevalence of ARMD was more in females as compared to males. A total of 54 (41.54%) subjects were men and 76 (58.46%) were women in the ARMD group, whereas 65 (50%) subjects were men and 65 (50%) subjects were women in the non-ARMD group. Higher levels of systolic blood pressure (SBP) were observed in 77 (59.13%) ARMD subjects in comparison to the non-ARMD group with 31 (23.85%) subjects (P<0.001), and similar results were observed for diastolic blood pressure (DBP). Out of 130 subjects diagnosed with ARMD, 68 (52.31%) subjects were found to be smokers as compared to 62 (47.69%) subjects who were nonsmokers. A higher number of smokers and alcohol drinkers suffered from ARMD in comparison to the non-ARMD group (P<0.001).

Remarkable differences (P<0.001) were seen for body mass index (BMI) as a risk factor, whereby 20 (15.38%) subjects with ARMD were obese (>29.9 kg/m^2^) in comparison to controls with 12 (9.23%) subjects who were obese. Fifty-seven (43.85%) subjects with ARMD were found to be in the range of 23-29.9 and 35 (26.92%) subjects in the non-ARMD group. More subjects having a sedentary lifestyle were present in the ARMD group (n=56, 43.08%) in comparison to controls (n=19, 14.62%). It is observable that those subjects who were exposed to sunlight for more than eight hours per day had more chances of having ARMD (P=0.0001).

Diastolic blood pressure augmented a 2.5-fold higher risk of ARMD, and smoking increases the risk by fourfold (P<0.0001). Univariate analysis suggested that BMI of >23 kg/m^2^ had an enhanced risk of ARMD development (OR: 0.33, 95%CI: 0.22-0.67, P=0.0007), which increased in the cases having BMI of ≥30 kg/m^2^ (OR: 0.38, 95%CI: 0.17-0.84, P=0.018). Table 1 summarizes the results of the univariate regression analysis of risk factors for ARMD.

**Table 1:** Univariate regression analysis of risk factors for ARMD NS: statistically not significant, S: statistically significant, LDL: low-density lipoprotein, HDL: high-density lipoprotein

Risk factors	Variables	ARMD	Non-ARMD	Total	Odds ratio (95%CI)	P-value
Age (years)	45-55	10 (7.69%)	15 (11.54%)	25 (9.62%)	Reference	
	56-65	63 (48.46%)	58 (44.62%)	121 (46.54%)	0.61 (0.25-1.47)	p=0.37^NS^
	66-75	49 (37.69%)	50 (38.46%)	99 (38.08%)	0.68 (0.27-1.66)	p=0.50^NS^
	76-85	8 (6.15%)	7 (5.38%)	15 (5.77%)	0.58 (0.16-2.12)	p=0.51^NS^
Gender	Male	54 (41.54%)	65 (50%)	119 (45.77%)	Reference	
	Female	76 (58.46%)	65 (50%)	141 (54.23%)	0.71 (0.43-1.16)	p=0.21^NS^
Smoking status	Nonsmokers	62 (47.69%)	103 (79.23%)	165 (63.46%)	Reference	
	Smokers	68 (52.31%)	27 (20.77%)	95 (36.54%)	0.23 (0.13-0.41)	p=0.0001^S^
Alcohol drinking	Nondrinkers	66 (50.77%)	85 (65.38%)	151 (58.08%)	Reference	
	Drinkers	64 (49.23%)	45 (34.62%)	109 (41.92%)	0.23 (0.13-0.41)	p=0.016^S^
Body mass index (kg/m^2^)	<23	53 (40.77%)	83 (63.85%)	136 (52.31%)	Reference	
	23-29.9	57 (43.85%)	35 (26.92%)	92 (35.38%)	0.33 (0.22-0.67)	p=0.0007^S^
	>29.9	20 (15.38%)	12 (9.23%)	32 (12.31%)	0.38 (0.17-0.84)	p=0.018^S^
Physical activity	Active	74 (56.92%)	111 (85.38%)	185 (71.15%)	Reference	
	Sedentary	56 (43.08%)	19 (14.62%)	75 (28.85%)	0.22 (0.12-0.71)	p=0.0001^S^
Total cholesterol levels (mg/dL)	≤200	103 (79.23%)	130 (100%)	233 (89.62%)	Reference	
	>200	27 (20.77%)	0 (0%)	27 (10.38%)	0.01 (0.0008-0.23)	p=0.0001^S^
LDL levels (mg/dL)	≤100	99 (76.15%)	125 (96.15%)	224 (86.15%)	Reference	
	>100	31 (23.85%)	5 (3.85%)	36 (13.85%)	0.12 (0.04-0.34)	p=0.0001^S^
Triglyceride levels (mg/dL)	≤150	103 (79.23%)	127 (97.69%)	230 (88.46%)	Reference	
	>150	27 (20.77%)	3 (2.31%)	30 (11.54%)	0.09 (0.02-0.30)	p=0.0001^S^
HDL levels (mg/dL)	<40	59 (45.38%)	6 (4.62%)	65 (25%)	Reference	
	≥40	71 (54.62%)	124 (95.38%)	195 (75%)	17.17 (7.05-41.79)	p=0.0001^S^
Systolic blood pressure (mmHg)	≤120	53 (40.77%)	99 (76.15%)	152 (58.46%)	Reference	
	>120	77 (59.13%)	31 (23.85%)	108 (41.54%)	0.21 (0.12-0.36)	p=0.0001^S^
Diastolic blood pressure (mmHg)	≤80	54 (41.54%)	89 (68.46%)	143 (55%)	Reference	
	>80	76 (58.46%)	41 (31.54%)	117 (45%)	0.32 (0.19-0.54)	p=0.0001^S^

β value suggested that every unit increase of alcohol enhanced the ARMD risk by 0.33±0.27 (β±SE) times, which is less compared to cigarette smoking, suggesting that cigarette smoking is the strongest independent risk factor associated with ARMD. Cigarette smoking appeared to be the strongest independent risk factor, which increased the risk of ARMD by four times (OR: 3.65, 95%CI: 2.05-6.50, P=0.0001) than those who did not smoke. β value suggested that every unit increase of smoking enhanced the ARMD risk by 1.44±0.29 (β±SE) times, which is much higher than alcohol drinking.

**Table 2:** Multivariate analysis of risk factors with ARMD NS: statistically not significant, S: statistically significant

Variables	β±SE	Odds ratio	95%CI	p-value
Age ≥ 56 years	0.20±0.46	1.23	0.49-3.03	0.651^NS^
DBP > 80 mmHg	0.85±0.28	2.35	1.34-4.13	0.003^S^
Alcohol	0.33±0.27	1.39	0.81-2.39	0.230^NS^
Smoking	1.44±0.29	4.25	2.40-7.52	0.0001^S^

## Discussion

This was a two-year-long hospital-based observational case-control study that included 260 subjects, of which 130 subjects have ARMD who were selected based on a drusen size of >63 microns on dilated fundoscopy and 130 subjects are non-ARMD subjects. The study conducted by Sharma et al. [11] has revealed that the prevalence of ARMD ranges from 1.8% in Karnataka to 47.8% in central Maharashtra. Kulkarni et al. [12] showed that the prevalence of ARMD was 1.38% with early ARMD at 1.34% and late ARMD at 0.37%.

According to the National Institute of Health, USA, two-thirds of the worst affected patients with ARMD are women. Age-related eye diseases study (AREDS) also showed that females are doubly affected by ARMD than males [13]. Females had significantly higher affection for developing ARMD than males in our study. A similar result was also noted in the studies of Kulkarni et al. [12], Pokharel et al. [14], and Arnarsson et al. [15].

Analyzing hypertension as a risk factor with ARMD, we have found that raised blood pressure is significantly associated with ARMD. In the univariate analysis carried out in this stud, we have found a correlation between ARMD and diastolic blood pressure, and this correlation was also found in the study by Sharma et al. [11]. Considering smoking as a major modifiable risk factor associated with ARMD, in this present study, we have found that smoking cigarettes is strongly correlated with ARMD (P<0.0001). The results are in accordance with various studies, such as those of Vashi et al. [16] and Sharma et al. [11], where they have found a significant correlation with cigarette smoking; however, the effects of passive smoking were not studied. Velilla et al. [17] showed that smoking has a 6.6 times higher risk in subjects with ARMD when compared to non-ARMD subjects, and this association increases as smoking pack years increases. The present study has shown the association of alcohol drinking with ARMD, which was found to be statistically significant.

Our results were consistent with the study by Sharma et al. [11] and Krishnaiah et al. [18], which observed that alcohol drinkers were at higher risk of developing ARMD as compared to nondrinkers (P<0.001). Similarly, a positive association was found in the Los Angeles Latino Eye Study between alcohol consumption and ARMD and was also observed in Melbourne Collaborative Cohort Study [19,20]. The Blue mountain study and POLA study showed a correlation of higher BMI with ARMD, which is in accordance with our results [21,22].

Reynolds et al. [23] showed that elevated LDL levels correlated with increased risk of ARMD in the rural population. Sharma et al. [11] documented the association of higher lipid levels and higher BMI with ARMD progression to late stages in comparison to non-ARMD. Seddon et al. [24] also showed similar results, demonstrating the correlation between ARMD and higher BMI, waist circumference, and waist/hip ratio. The study by Knudtson et al. [25] proved that the association of ARMD with physical activity is independent of other confounders such as BMI, smoking, and alcohol. They have demonstrated a protective effect of an active lifestyle on ARMD as the age increases in comparison to a population having a sedentary lifestyle.

Vashi et al. [16] also showed that more sunlight exposure increases the risk of developing late ARMD twofold. Tomany et al. [26] studied the 10-year incidence of ARMD with sunlight exposure and stated that people with more than five hours of light exposure have a higher risk of developing ARMD and that the use of hats and sunglasses showed a little protective effect on drusen. Cigarette smoking is considered one of the strongest risk factors correlated with ARMD progression, and it is 4.25 times compared to nonsmokers. However, the effect of quitting smoking has not been demonstrated in this study, but it suggests that every year, it increases risk by 1.4 units when measured in terms of pack years. Diastolic blood pressure of more than 80 mmHg after smoking as a risk factor is significantly associated with ARMD progression, suggesting a 2.35 times risk of developing as compared to subjects with normal diastolic blood pressure. Elevated blood pressure can increase by 0.85 units every year if it remains untreated.

This study has a few limitations in that it has a small sample size, so larger-scale community studies would be beneficial for making the findings more stable. There was no follow-up period, so the correlation of risk factors in the long run was not taken into consideration. The results may have been underestimated or overestimated as we have not performed optical coherence tomography (OCT)/fundus fluorescein angiography (FFA) to confirm the diagnosis.

## Conclusions

ARMD is a complex disease that causes irreversible visual loss and is associated with multiple modifiable and non-modifiable risk factors, and treatment options are limited in advanced stages. Diagnosis of this disease usually occurs in later stages when visual loss already has occurred, and no effective treatment can be given. So, making early diagnosis is the key to prevent this disease. The various modifiable risk factors associated with ARMD, if detected early and controlled, could save patients’ visual acuity without progressing to late stages. Thus, this article stress on identifying patients with specific risk factors so that we can prevent the progression of this disease to later stages. This study showed the strongest association of cigarette smoking with ARMD, followed by diastolic blood pressure and alcohol drinking, and body mass index has a weak association among modifiable risk factors.
